# Paraneoplastic cerebellar degeneration as initial presentation of renal cell carcinoma

**DOI:** 10.1186/s40673-019-0102-9

**Published:** 2019-07-10

**Authors:** Sara M. Souza, Barbara O. Santos, Isadora C.A. Sodré, Ana Luiza P. Oliveira, Diogo Terrana, Mariana Spitz

**Affiliations:** Neurology Service, Pedro Ernesto University Hospital, State University of Rio de Janeiro, Blv 28 de setembro, 77 – Vila Isabel, Rio de Janeiro, 20551-030 Brazil

**Keywords:** Paraneoplastic, Ataxia, Cerebellar degeneration

## Abstract

**Background:**

Paraneoplastic cerebellar degeneration is usually associated with gynecological and breast cancer, lung cancer, and Hodgkin’s lymphoma. Renal cell carcinoma has rarely been described as an underlying malignancy in these cases.

**Case presentation:**

We report the case of a 75 year-old woman who develop cerebellar ataxia following a respiratory viral infection. During investigation, around 1 year afterward, she noticed constitutional symptoms suggestive of malignancy. Renal carcinoma was found and the hypothesis of paraneoplastic cerebellar degeneration was considered.

**Conclusions:**

As no specific antineuronal antibodies have been described in the setting of renal cell carcinoma, paraneoplastic cerebellar degeneration should be considered when the tumor is detected and other causes are excluded. Immunotherapy should be prescribed as soon as possible.

## Introduction

Paraneoplastic cerebellar degeneration belongs to a group of neurologic syndromes in which symptoms are indirectly caused by an underlying malignancy. Cerebellar signs and symptoms are not related to a metastatic disorder; instead, the mechanism of injury is autoimmune [1]. It can be secondary to any cancer, although the most common are gynecological and breast cancer, lung cancer, and Hodgkin’s lymphoma [2]. The neurologic symptoms may precede the diagnosis of cancer by several years [3]. Hens et al., for example, reported a paraneoplastic cerebellar syndrome that began 6 years previous to the detection of a renal cell carcinoma [2].

## Case presentation

A 75-year-old female, with a past medical history of hypertension, developed progressive cerebellar ataxia a week after a respiratory viral infection. She reported dysarthria and gait unsteadiness. Her symptoms worsened for about 2 weeks and then remained stable. She was admitted to our service 1 year after symptoms onset, and she had been previously diagnosed with parainfectious cerebellar ataxia. She also reported a 6-month history of daily nausea and vomiting, weight loss of 66 pounds, and distal four limbs paresthesias.

**Fig. 1:**
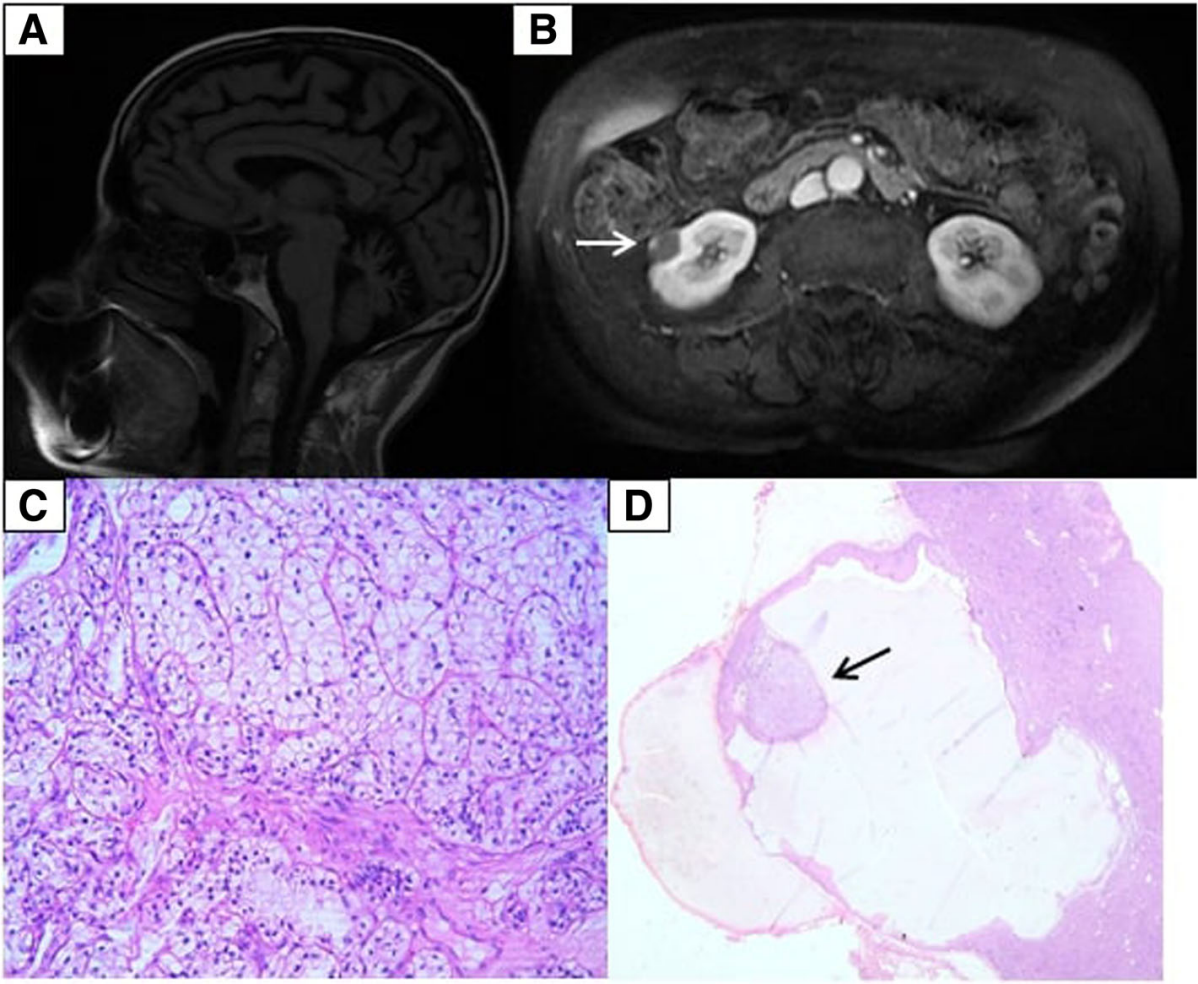
**a** Sagittal T1-weighted brain MRI with cerebellar atrophy. **b** Axial T2- weighted abdominal MRI with oval exophytic hypodense mass, with blood content, and focal parietal thickening area of nodular aspect (white arrow), measuring 1.6 X1.1 cm. **c** and **d** Renal cell carcinoma (black arrow in **d**), clear cell variant, grade 2 Fuhrman nuclear, predominantly cystic, encapsulated. The neoplasm does not invade the renal capsule. No vascular invasion was observed. (**c**: 40x/0.65 HE. **d**: 4x/0.1 HE)

Neurological examination revealed severe dysarthria, bilateral horizontal nystagmus, pronounced ataxia, and bilateral dysdiadochokinesia. Although she was unable to stand or walk unattended, there was no muscle weakness. She had generalized hypotonia. Deep tendon reflexes were normal in the upper limbs, and absent in the lower limbs. There was distal pinprick hypoesthesia in both upper and lower limbs, with reduced proprioception and vibration sense in the lower limbs. Cognition was preserved and there were no mood complaints. The patient denied bowel and bladder dysfunction. Blood exams were unremarkable. Electromyography revealed four limbs severe axonal sensitive polyneuropathy, with mild motor involvement. She was prescribed high-dose intravenous methylprednisolone 1 g qd for 3 days based on the hypothesis of an auto-immune condition, but there was no clinical improvement. Serological screening was requested and included protein electrophoresis, anti-tissue transglutaminase IgG and IgA, anti-gliadin IgA, IgG, IgM, and anti-GAD and the results were non-reactive. Paraneoplastic antibodies panel, which was also negative, comprised anti-HU, anti-YO, anti-RI, anti-amphiphysin, anti-CV2, anti-MA2, anti-MGT30, AGNA and anti-recoverin. Lumbar puncture was performed 1 year after symptoms onset, and spinal fluid analysis showed 4 cells, protein of 28 mg/dL and glucose of 70 mg/dL. Brain MRI (Fig. 1a) displayed cerebellar atrophy whereas the cerebral cortex had no abnormalities. Further tests were requested to evaluate the frequent vomiting, which persisted during the entire period of hospital stay. Abdominal MRI (Fig. 1b) showed a right exophytic heterogeneous renal mass. Radiology determined that it was category III in the Bosniak Classification for complex renal cysts, suggesting a high risk of malignancy [4]. The tumor was subsequently resected, and the histopathologic analysis revealed a clear cell renal carcinoma (Fig. 1c and d). The lesion had a cystic greater than nodular component, which is atypical. Predominantly cystic lesions suggest fast-growing tumors [5].

On 6-month follow up after tumor extraction, there was complete resolution of gastrointestinal symptoms, and partial dysarthria improvement. Ataxia, however, remained stable. The patient was referred to the Movement Disorders Outpatient Clinic. There were no further therapeutic interventions after hospital discharge.

## Discussion

The patient herein described had paraneoplastic cerebellar degeneration as the first manifestation of a renal carcinoma, which is a very unusual association. Although there was an infection preceding symptom onset, gastrointestinal symptoms and significant weight loss made the diagnosis of post-viral cerebellar syndrome less likely.

The presentation of renal cell carcinoma-associated paraneoplastic neurological syndrome has been described as a rare condition. Another manifestations besides cerebellar degeneration have been reported, such as opsoclonus-myoclonus syndrome and frontal lobe disorder [6, 7]. .In contrast to other paraneoplastic syndromes, such as lung, breast or ovarian cancer, in which specific antineuronal antibodies have been identified, no antineuronal antibodies have been described in renal cell carcinoma. However, there is strong evidence of a causative association between neurological lesion and an underlying immune-mediated tumor process [7].

This case highlights the need to consider paraneoplastic cerebellar degeneration in patients presenting with gradually progressive cerebellar ataxia, not explained by other causes. We also emphasize that although anti-neuronal antibodies reinforce the diagnosis of a paraneoplastic syndrome, the fact that they are not detected does not exclude the diagnosis. According to the classification by Graus et al, in this case a paraneoplastic disorder was classified as possible due to neurological symptoms and the concomitant tumor, but the anti-neuronal antibodies investigated were negative [8]. Despite the benefits of immunotherapy for paraneoplastic disorders in several reported cases [9], we consider that in our case the delay between clinical presentation onset and immunotherapy prescription led to a suboptimal clinical response. The failure to obtain the correct diagnosis of paraneoplastic syndromes may allow patients to progress to more advanced cancer stages.

## Conclusions

As no specific antineuronal antibodies have been described in the setting of renal cell carcinoma, paraneoplastic cerebellar degeneration should be considered when the tumor is detected and other causes are excluded. Immunotherapy should be prescribed as soon as possible.

## Data Availability

The datasets used and/or analysed during the current study are available from the corresponding author on reasonable request.
